# Real-world assessment of multipolar and point-by-point mapping for premature ventricular contraction ablation

**DOI:** 10.1093/europace/euae148

**Published:** 2024-05-31

**Authors:** Pedro A Sousa, Johanna Tonko, Dagmara Dilling-Boer, Sérgio Barra, Anna-Sophie Eberl, Borka Pezo, Nuno Cortez-Dias, Ziad Khoueiry, Paulo Medeiros, Moisés Rodríguez-Mañero, Ana Lebreiro, Mariana Pereira, Luís Puga, Daniel Scherr, Natália António, Afonso Ferreira, Carolina Saleiro, Philippe Lagrange, Luis Adão, Joao de Sousa, Luís Elvas, Mário Oliveira, Lino Gonçalves, John Silberbauer

**Affiliations:** Pacing & Electrophysiology Unit, Cardiology Department, Coimbra’s Hospital and University Center, Morada: Praceta Prof Mota Pinto, 3000-075 Coimbra, Portugal; Cardiology Department, Sussex Cardiac Centre, Brighton, UK; Institute of Cardiovascular Science, University College London, London, UK; Cardiology Department, Hartcentrum Hasselt, Hasselt, Belgium; Cardiology Department, Hospital da Luz Arrábida, V. N. Gaia, Portugal; Division of Cardiology, Department of Medicine, Medical University of Graz, Graz, Austria; Cardiology Department, University Hospital Centre Zagreb, Zagreb, Croatia; Arrhythmia Unit, Cardiology Department, Santa Maria University Hospital, Lisbon Academic Medical Center, Lisbon, Portugal; Cardiology Department, Clinique Saint Pierre, Perpignan, France; Cardiology Department, Centro Hospitalar de Entre Douro e Vouga, Santa Maria da Feira, Portugal; Cardiology Department, University Center Hospital of Santiago, Santiago de Compostela, Spain; Cardiology Department, University Hospital Center of São João, Porto, Portugal; Biosense Webster, Portugal; Cardiology Department, Centro Hospitalar de Entre Douro e Vouga, Santa Maria da Feira, Portugal; Division of Cardiology, Department of Medicine, Medical University of Graz, Graz, Austria; Pacing & Electrophysiology Unit, Cardiology Department, Coimbra’s Hospital and University Center, Morada: Praceta Prof Mota Pinto, 3000-075 Coimbra, Portugal; Faculty of Medicine, ICBR, University of Coimbra, Coimbra, Portugal; Cardiology Department, Santa Marta Hospital, Lisboa, Portugal; Arrhythmia Unit, Cardiology Department, Santa Maria University Hospital, Lisbon Academic Medical Center, Lisbon, Portugal; Pacing & Electrophysiology Unit, Cardiology Department, Coimbra’s Hospital and University Center, Morada: Praceta Prof Mota Pinto, 3000-075 Coimbra, Portugal; Cardiology Department, Clinique Saint Pierre, Perpignan, France; Cardiology Department, University Hospital Center of São João, Porto, Portugal; Arrhythmia Unit, Cardiology Department, Santa Maria University Hospital, Lisbon Academic Medical Center, Lisbon, Portugal; Pacing & Electrophysiology Unit, Cardiology Department, Coimbra’s Hospital and University Center, Morada: Praceta Prof Mota Pinto, 3000-075 Coimbra, Portugal; Cardiology Department, Santa Marta Hospital, Lisboa, Portugal; Pacing & Electrophysiology Unit, Cardiology Department, Coimbra’s Hospital and University Center, Morada: Praceta Prof Mota Pinto, 3000-075 Coimbra, Portugal; Faculty of Medicine, ICBR, University of Coimbra, Coimbra, Portugal; Cardiology Department, Sussex Cardiac Centre, Brighton, UK

**Keywords:** Multielectrode mapping catheter, Point-by-point mapping, Premature ventricular contraction, Left-sided premature ventricular contraction, Catheter ablation

## Abstract

**Aims:**

We aimed to assess the acute and midterm efficacy of premature ventricular contraction (PVC) ablation guided by multielectrode and point-by-point (PbP) mapping.

**Methods and results:**

This is a retrospective, international multicentre study of consecutive patients referred for PVC ablation in 10 hospital centres from January 2017 to December 2021. Based on the mapping approach, two cohorts were identified: the ‘Multipolar group’, where a dedicated high-density mapping catheter was employed, and the ‘PbP group’, where mapping was performed with the ablation catheter. Procedural endpoints, safety, and acute (procedural) and midterm efficacies were assessed. Of the 698 patients included in this study, 592 received activation mapping [46% males, median age of 55 (41–65) years]—248 patients in the Multipolar group and 344 patients in the PbP group. A higher number of activation points [432 (217–843) vs. 95 (42–185), *P* < 0.001], reduced mapping time (40 ± 38 vs. 61 ± 50 min, *P* < 0.001), and shorter procedure time (124 ± 60 vs. 143 ± 63 min, *P* < 0.001) were reported in the Multipolar group. Both groups had high acute success rates (84.7% with Multipolar mapping vs. 81.3% with PbP mapping, *P* = 0.63), as well as midterm efficacy (83.4% vs. 77.4%, *P* = 0.08), with no significant differences in the risk of adverse events (6.0% vs. 3.5%, *P* = 0.24). However, for left-sided PVC ablation specifically, there was a higher midterm efficacy in the Multipolar group (80.7% vs. 69.5%, *P* = 0.04), with multipolar mapping being an independent predictor of success [adjusted OR = 2.231 (95% CI, 1.476–5.108), *P* = 0.02].

**Conclusion:**

The acute and midterm efficacies of PVC ablation are high with both multipolar and PbP mapping, although the former allows for quicker procedures and may potentially improve the outcomes of left-sided PVC ablation.

## Graphical Abstract

**Graphical Abstract:**
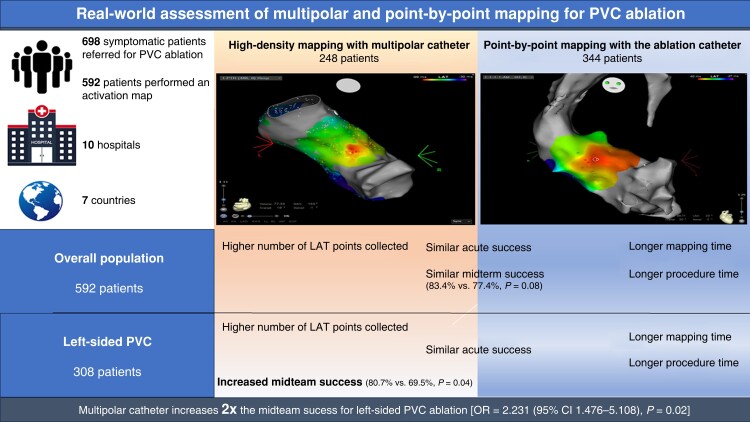

**What’s new?**

- This international multicentre study is the largest study assessing two different mapping strategies for premature ventricular contraction (PVC) ablation in a large real-world cohort of patients: one guided by high-density mapping using a multipolar catheter vs. one employing point-by-point mapping with the ablation catheter.
- Multipolar mapping allowed a detailed activation map and faster mapping and procedure times.
- Overall, both strategies present high acute and midterm efficacies for PVC ablation.
- For the specific subgroup of patients having left-sided PVC ablation, the use of the multipolar mapping may be associated with higher freedom from ventricular ectopies during follow-up, with multipolar mapping being an independent predictor of success.

## Background

Frequent premature ventricular contractions (PVCs) may be associated with an increased risk of congestive heart failure and mortality in specific patients.^1^ Catheter ablation is an established treatment to abolish and/or reduce PVC burden, improve symptoms, and prevent and/or reverse PVC-induced cardiomyopathy.^2,3^ Recently, the combined use of the PentaRay^TM^ (Biosense Webster, Inc.) mapping catheter with the pattern matching filter (PMF) software (Biosense Webster, Inc.) was shown to improve not only the level of detail, accuracy, and reliability of the local activation time (LAT) map, but also the success rates of PVC ablation when compared to point-by-point (PbP) mapping with the ablation catheter.^4–6^ However, in these studies, multielectrode mapping was performed in a few highly specialized centres. The benefit of the widespread use of multielectrode mapping for PVC ablation remains unclear.

This international multicentre study aimed to assess procedural characteristics and acute and midterm success rates of PVC ablation guided by a multipolar catheter or PbP mapping in a large cohort of patients referred for this procedure.

## Methods

### Study design and setting

This was a retrospective, international multicentre study. Consecutive patients with frequent symptomatic PVC referred for catheter ablation from January 2017 to December 2021 in 10 different hospital centres were screened. Baseline demographics, medical records, including cardiac imaging, electrocardiograms, and rhythm monitoring results, procedural characteristics, and outcomes of catheter ablation, as well as follow-up data were reviewed and analysed.

Patients who underwent PVC ablation guided by ventricular activation mapping (LAT, local activation map) were included and allocated to separate cohorts based on the type of mapping catheter employed for activation mapping: a multipolar mapping catheter (Multipolar group) or an ablation catheter (PbP group). Acute and midterm results were assessed, as well as procedural endpoints.

The study was approved by the local institutional ethics committee (OBS.SF.032–2022). No patient or public was involved in the design or execution of the study.

### Patient eligibility criteria

All patients referred for PVC ablation were eligible for inclusion in the study. Patients were ultimately included if the ablation strategy was guided by LAT mapping, and procedural outcome and follow-up data were available for review. The necessary diagnostic workup to rule out structural heart disease was performed at the discretion of physicians in the enrolling hospitals. Generally, significant coronary artery disease was ruled out by cardiac angiography or stress testing in cases of left ventricular systolic dysfunction. Structural heart disease included ischaemic heart disease, congenital heart disease, hypertrophic cardiomyopathy, dilated cardiomyopathy, myocarditis, arrhythmogenic right ventricular cardiomyopathy, or severe valvular disease.

Exclusion criteria included reduced (<1 PVC/min) and/or absence of PVC during the electrophysiological study precluding the acquisition of a reliable activation map^7^ and/or an ablation strategy based on pace mapping alone as per operator preference.

### Procedure details

All participating centres are high-volume electrophysiology departments that routinely perform PVC ablation, with all procedures performed by or under the supervision of an experienced electrophysiologist. Higher-volume PVC ablation centres were defined as those performing more than 10 PVC ablations per year.

**Figure 1:**
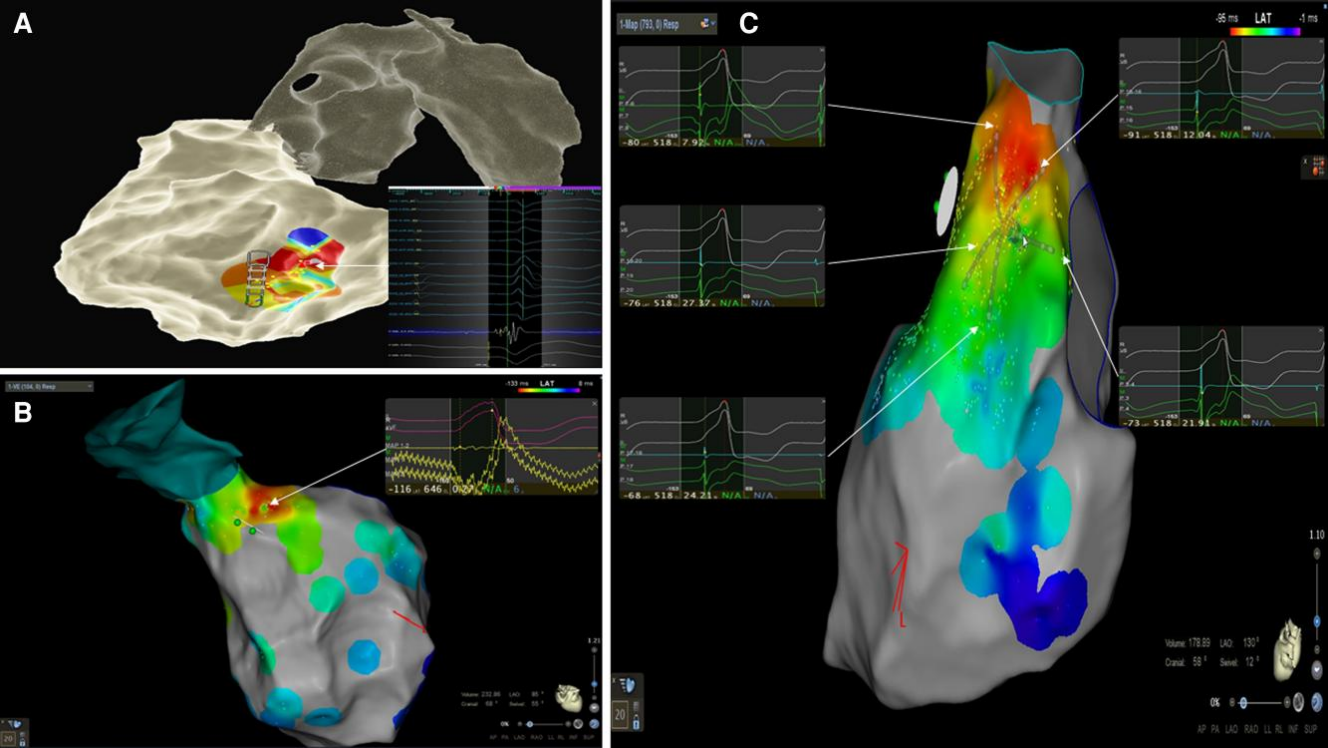
(*A*) Left PVC activation map performed with the ablation catheter (Ensite Precision^TM^), resulting in few LAT points collected and therefore less detailed and potentially less accurate. (*B*) PVC activation map with the earliest activation point located in the aorto-mitral continuity. The activation map was performed with the ablation catheter (Biosense Webster) resulting in 104 LAT points. The absence of a catheter for unipolar signal resulted in a highly fractionated unipolar electrogram, sometimes confusing the automatic annotation by the Wavefront activation software, resulting in some incorrect LAT annotations. (*C*) Activation mapping performed with a multipolar mapping catheter (PentaRay^TM^, Biosense Webster) and a catheter for unipolar signal. This high-density activation mapping included 793 points, resulting in a very detailed and accurate map. It highlights the benefits of the multipolar catheter—the possibility of acquiring multiple LAT points with a single PVC and the capability of covering a large area. Also, by using a dedicated catheter for unipolar signal, we observed a clear unipolar endocardial electrogram that resulted in correct annotation by the Wavefront annotation software.

Patients underwent PVC ablation procedures according to the strategy of each enrolling centre. In general, all AADs were interrupted at least five half-lives before the procedure (except for amiodarone, which was interrupted at least 2 weeks before the procedure). A retrograde approach was initially used for left-sided procedures, but access through transseptal puncture was also obtained whenever necessary. Intravenous heparin was administered to maintain an activated clotting time ≥ 300 s for left-sided procedures. With reduced (<1 PVC/min) or absent PVC during the electrophysiological study, intravenous administration of isoproterenol or programmed ventricular stimulation was performed. If the number of PVCs remained too low to allow LAT mapping, the patient was excluded from the study.^7^ All cases were performed with a 3D mapping system, which could be complemented with intracardiac echocardiography (ICE) or image integration. The choice to perform mapping with a multipolar catheter or with the ablation catheter was left to the physician’s discretion. Patients were allocated to the Multipolar or PbP groups according to which mapping strategy was employed by the physician at the start of the case. Patients were excluded from the study when a change of mapping strategy/catheter occurred during the procedure. However, an exception was made for those cases when the physician switched from multipolar mapping to mapping with the ablation catheter due to frequent mechanically induced PVCs precluding the former strategy (in which case, this would need to be explicitly reported; otherwise, the patient would be excluded from the study). The use of an additional catheter for obtaining a clean unipolar signal typically obtained through positioning an electrode in the inferior vena cava was also left to the physician’s discretion. This would avoid extrapolation from ECG Wilsons triangle and noise (for example in the CARTO system, automatic signal annotation is performed using Wavefront activation software, which analyses and incorporates bipolar signals and the corresponding negative onset of the unipolar signal)^8,9^ (*Figure 1*). Physicians were allowed to perform pace mapping or manual verification of LAT points before ablation.

Ablation was always performed with an irrigated catheter. Generally, the power setting was set at 20 W, and this could be titrated up to 50 W according to location. The use of a sheath for increasing the stability of the ablation catheter was left to the physician’s discretion. During the waiting period, intravenous administration of isoproterenol or programmed ventricular stimulation was used to confirm the disappearance of the PVC, and if there was a recurrence during this period, ablation was repeated.

### Study endpoints

The primary endpoint of the study was midterm efficacy defined as a reduction of the PVC burden of at least 80% in a 24 h Holter carried out at least 3 months after the index procedure.^10^

Secondary endpoints included assessment of acute success, procedural data, adverse events within 1 month, and predictors of recurrence. Acute success was defined as the complete abolition of the clinical PVC at the end of the procedure. Procedural data included the number of activation points acquired, mapping, ablation, and total procedural times. Adverse events included any moderate or major complications. Minor adverse events included femoral arterial pseudoaneurysm or arteriovenous femoral fistula not requiring intervention/surgery, pericardial effusion managed conservatively, and second- or third-degree AV block. Major complication included death, stroke, or other systemic emboli, ventricular fibrillation, or pulseless ventricular tachycardia, infective endocarditis, cardiac tamponade, vascular injury requiring transfusion, or vascular surgery and damage to the valves or coronary arteries.

A complementary analysis was carried out to assess the outcomes of ablation guided by multipolar or PbP mapping according to the ventricular chamber where ablation was performed. To facilitate this analysis, patients were divided into two groups, right-sided or left-sided PVCs, with the latter including PVCs originating from both the left ventricle and the aortic root. The locations where RF was successful in abolishing the PVC focus determined whether it was considered right or left-sided (although we acknowledge that, on occasion, PVC location may be intramural and requiring ablation from both sides). PVCs from the LV summit, great cardiac vein, or septal vein were also considered as being left-sided.

### Follow-up

Assessment of the primary endpoint was made following at least 3 months of the index procedure. The use of AAD after the ablation procedure was left to the physician’s discretion. A 12-lead electrocardiogram (ECG) and a 24 h Holter were performed 3 months post-ablation. PVC recurrences and burden were obtained from patients’ files in each centre, including clinical diaries and results of the 24 h Holter. In case of previous LV dysfunction, assessment of LVEF by echocardiography was also performed to evaluate the improvement of LVEF. Data on complications potentially related to the procedure were obtained from procedure reports and clinical diaries.

### Statistical analysis

Categorical variables were expressed in frequencies and percentages, and continuous variables were expressed as mean ± standard deviation or median and interquartile range (IQR) for variables with or without normal distribution, respectively. The X^2^ test was used to assess differences between categorical variables, and the Student's *t*-test or the Wilcoxon test was used to compare continuous variables with or without normal distribution, respectively. Normality of distribution of continuous variables was assessed using the Kolmogorov–Smirnov test. Multivariate Cox proportional regression analysis was used to identify predictors of ventricular arrhythmia recurrence, with adjustment on any variables differing between groups in univariate analysis. A sensitive analysis of left-sided PVC ablations was performed to evaluate whether multipolar mapping improved the outcome in this subset of patients. An additional analysis was also performed to identify any possible differences concerning the primary endpoint between higher- and lower-volume centres. A *P* value < 0.05 was considered statistically significant. Statistical analysis was performed using IBM SPSS Statistics Version 25 (IBM, Armonk, New York) software.

## Results

**Figure 2:**
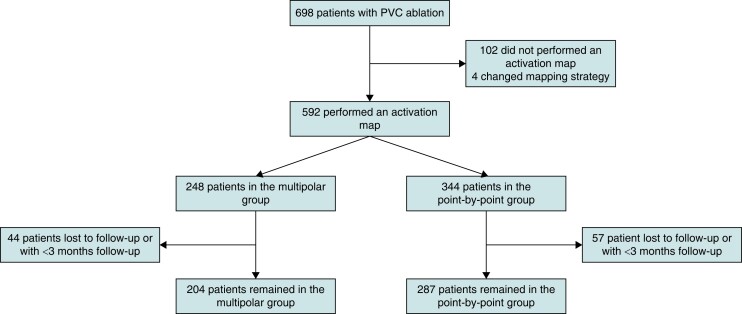
Study flowchart protocol.

Of the 698 patients initially considered for this study, 106 were excluded for not receiving activation mapping (102) or having the mapping strategy modified during the case (4), as per study protocol (*Figure 2*). The final study sample included 592 patients [46% males, median age of 55 (41–65) years]—248 patients in the Multipolar group and 344 patients in the PbP group. The main baseline characteristics are outlined in *Table 1*. Patients in the Multipolar group were more often male (51% vs. 43%, *P* = 0.03) and slightly older (55 vs. 53 years, *P* = 0.05), and there was a trend towards more frequent structural heart disease (*P* = 0.09) and presence of late gadolinium enhancement (*P* = 0.09) on cardiac magnetic resonance. There were no differences regarding the use of AAD (*P* = 0.23), previous history of PVC ablation (*P* = 0.27), or number of PVCs (*P* = 0.72) (see Supplementary material online, *Table S1*).

**Table 1:** Baseline characteristics

	All patients (*n* = 592)	Multipolar group (*n* = 248)	Point-by-point group (*n* = 344)	*P* value
Age, years [median (IQR)]	54 (41–65)	55 (42–67)	53 (39–63)	0.05
Male gender, *n* (%)	273 (46)	127 (51)	146 (42)	0.03
Previous PVC ablation, *n* (%)	67 (11.3)	24 (9.7)	43 (12.5)	0.27
Structural heart disease, *n* (%)	173 (29.2)	83 (32.5)	90 (26.2)	0.09
CMR, *n* (%)	261 (44.1)	106 (42.7)	155 (45.1)	0.22
LGE, *n* (%)	51 (8.6)	27 (10.9)	24 (7.0)	0.09
≥2 PVC ectopies, *n* (%)	129 (21.8)	50 (20.2)	79 (23.0)	0.17
Under AAD, *n* (%)	419 (70.8)	169 (68.1)	250 (72.7)	0.23
LVEF, % [median (IQR)]	55 (50–60)	55 (45–60)	57 (50–60)	0.002
PVC number, median (IQR)	20 197 (12 147–29 275)	20 086 (11 792–30 422)	20 700 (14 185–28 297)	0.72
% PVC, median (IQR)	20 (12–30)	23 (13–32)	19 (12–29)	0.04
Left-sided origin, *n* (%)	308 (52)	153 (61.7)	155 (45.3)	<0.001

AAD, antiarrhythmic drug; CMR, cardiac magnetic resonance; LGE, late gadolinium enhancement; LVEF, left ventricle ejection fraction; PVC, premature ventricular contraction

In both groups, the RVOT was the most common PVC location, followed by the left ventricular outflow tract and aortic coronary cusps (see Supplementary material online, *Table S2*). A left-sided PVC origin was more frequent in the Multipolar group (61.7% vs. 45.3%, *P* < 0.001), with increased PVC origin in the coronary cusps (12.5% vs. 8.7%, *P* = 0.002) and in the LV summit (9.7% vs. 4.4%, *P* = 0.004) and a trend towards a higher PVC ablation in the papillary muscles (5.6% vs. 2.6%, *P* = 0.06).

### Procedural characteristics

A detailed assessment of the two groups regarding procedural data is provided in *Table 2*. The majority of centres used the CARTO system, with PentaRay being the most frequently used multielectrode catheter (60.9% PentaRay^TM^ vs. 2% Lasso^TM^ vs. 8.5% Decanav^TM^ vs. 23% Advisor HD Grid^TM^ vs. 1.2% Array^TM^ vs. 4.4% IntellaMapOrion^TM^, *P* < 0.001). In the Multipolar group, ICE (13% vs. 0.9%, *P* < 0.001) and cardiac image integration (26.2% vs. 8.4%, *P* < 0.001) were more frequently used, whereas insertion of a dedicated catheter for unipolar referencing (49.2% vs. 64.5%, *P* < 0.001) and coronary angiography (8.1% vs. 29.4%, *P* < 0.001) were less commonly performed.

**Table 2:** Procedural parameters

	All patients (*n* = 592)	Multipolar group (*n* = 248)	Point-by-point group (*n* = 344)	*P* value
Mapping system, *n* (%)				<0.001
CARTO, *n* (%)	480 (81.1)	177 (71.4)	303 (88.1)	
NavX/Ensite, *n* (%)	99 (16.7)	60 (24.2)	39 (11.3)	
Rhythmia, *n* (%)	13 (2.2)	11 (4.4)	2 (0.6)	
Software for automatic annotation				
PMF Biosense, *n* (%)	389 (81)	149 (84.1)	240 (79.2)	0.09
AutoMap Abbott, *n* (%)	93 (93.9)	59 (98.3)	34 (87.2)	<0.001
Favourite beat Boston, *n* (%)	11 (84.6)	10 (91)	1 (50)	0.08
Catheter for unipolar signal, *n* (%)	344 (58.1)	122 (49.2)	222 (64.5)	<0.001
Image integration, *n* (%)	94 (15.9)	65 (26.2)	29 (8.4)	<0.001
ICE, *n* (%)	35 (5.9)	32 (12.9)	3 (0.9)	<0.001
Retrograde access, *n* (%)	247 (41.7)	107 (43.1)	140 (40.7)	0.42
Coronary angiography, *n* (%)	121 (20.4)	20 (8.1)	101 (29.4)	<0.001
Use of ablation tools (AI, LSI, AutoTag), *n* (%)	285 (48.1)	211 (85.1)	74 (21.5)	<0.001
Patients with few PVCs during the procedure, *n* (%)	88 (14.9)	35 (14.1)	53 (15.4)	0.67
Pace mapping, *n* (%)	398 (67.2)	126 (50.8)	272 (79.1)	<0.001
Points acquired, *n* [median (IQR)]	165 (71–452)	432 (217–843)	95 (42–185)	<0.001
Manual verification of the points, *n* (%)	390 (65.9)	94 (37.8)	296 (86.0)	<0.001
Power, Watts (mean ± SD)	37 ± 8	39 ± 9	36 ± 7	<0.001
Mapping time, min (mean ± SD)	52 ± 44	40 ± 38	61 ± 50	<0.001
CF sensing catheter, *n* (%)	503 (85.0)	216 (87.1)	287 (83.4)	0.44
Ablation time, s (mean ± SD)	639 ± 612	570 ± 448	732 ± 770	0.03
Procedure time, min (mean ± SD)	136 ± 62	124 ± 60	143 ± 63	<0.001

AI, ablation index; CF, contact force; ICE, intracardiac echocardiography; PMF, pattern matching filter; PVC, premature ventricular contraction; LSI, lesion size index

The median number of electrograms (EGM) per map was 432 (217–843) during an average mapping time of 40 ± 38 min in the Multipolar group compared with 95 (42–185) (*P* < 0.001) during 61 ± 50 min (*P* < 0.001) in the PbP group (*Table 2* and *Figure 1*). In two cases (both right-sided PVC), there was a need to shift from the PentaRay^TM^ multipolar catheter to the ablation catheter due to very frequent mechanically induced PVC. Additional pace mapping (50.8% vs. 79.1%, *P* < 0.001) and manual confirmation of the earliest points (39% vs. 87.1%, *P* < 0.001) were less commonly reported in the Multipolar group.

**Figure 3:**
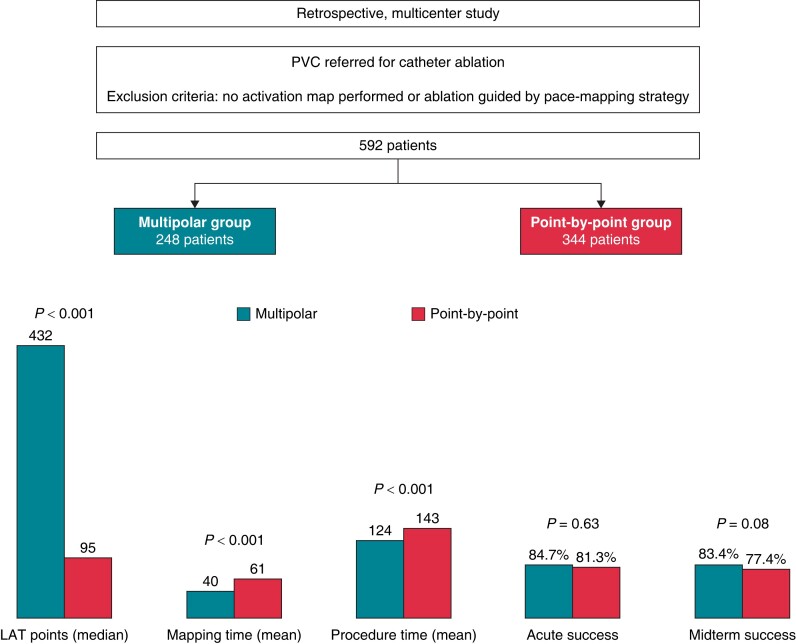
Procedural data and acute and midterm outcomes in the general population.

Ablation was attempted in all procedures, with ethanol ablation performed in 6 (2.4%) cases in the Multipolar group and 1 case (0.3%) in the PbP group. The RF (570 vs. 732 s, *P* = 0.03) and total procedure times (124 ± 60 vs. 143 ± 63 min, *P* < 0.001) were significantly lower in the Multipolar group (*Table 2* and *Figure 3*).

### Acute success and safety

A high acute success rate was seen regardless of the mapping strategy (84.7% with Multipolar mapping vs. 81.3% in the PbP group, *P* = 0.63), despite the PVC recurrence during the waiting period in almost one-fifth of patients (22.5% overall; 17.1% in Multipolar group vs. 24.2% with PbP mapping, *P* = 0.25) (*Figure 3* and Supplementary material online, *Figure S1*). After adjusting for all predictors of acute success in univariate analysis, PVC recurrence during the waiting time was the only independent predictor of acute recurrence [OR = 0.023 (95% CI, 0.008–0.067), *P* < 0.001] (see Supplementary material online, *Table S3*).

The overall complication rate was 4.6%, with a non-significant trend for a higher rate in the Multipolar group (6.0% vs. 3.5% in the PbP group, *P* = 0.24) likely unrelated with the mapping strategy itself. No deaths occurred. In the Multipolar group, major complications occurred in 6 patients (2.4%)—one stroke due to air embolism resolved without any sequelae after hyperbaric chamber, two cardiac tamponades, two pseudoaneurysms required thrombin injection, and one coronary artery dissection with stent implantation. Conversely, in the PbP group, major adverse events occurred in 6 patients (1.7%)—one case of retinal thrombosis, one cardiac tamponade, and three pseudoaneurysms, one requiring surgery and two requiring thrombin injection. All complications are detailed in Supplementary material online, *Table S4*.

### Midterm follow-up

Median follow-up was 8 (3–22) months, with 491 patients completing a minimum follow-up of 3 months. There was no significant difference in the follow-up duration between groups (*P* = 0.22) (*Figure 2*). Overall freedom from ventricular arrhythmia was 80% at 3 months, without significant differences between groups (83.4% in the Multipolar group vs. 77.4% in the Control group, *P* = 0.08) (*Figure 3*). AADs were used during follow-up in one-third of patients in both groups, with beta-blockers being the most frequently used. After adjustment on all univariate predictors of the primary outcome the use of a catheter for unipolar signal [OR = 2.652 (95% CI, 1.275–5.518), *P* = 0.009] was independently associated with freedom from recurrent PVC during follow-up (*Table 3*). Recurrence during the waiting period was associated with increased PVC recurrence during midterm follow-up [OR = 0.330 (0.162–0.672), *P* = 0.002].

**Table 3:** Predictors of PVC recurrence (>20% PVC burden at midterm follow-up)

	All patients (*n* = 491)	PVC reduction <80% (*n* = 98)	PVC reduction ≥ 80% (*n* = 393)	Univariate model		Multivariate model	
				OR (95% CI)	*P* value	OR (95% CI)	*P* value
Male gender, *n* (%)	226 (46)	47 (48)	179 (46)	1.275 (0.626–2.597)	0.50		
Age, years [median (IQR)]	54 (41–65)	53 (36–62)	53 (39–63)	1.004 (0.989–1.020)	0.57		
Previous PVC ablation, *n* (%)	57 (11.6)	14 (14.3)	43 (10.9)	0.879 (0.419–1.844)	0.46		
Structural heart disease, *n* (%)	136 (27.7)	41 (41.8)	95 (24.2)	0.435 (0.248–0.763)	0.03	0.580 (0.281–1.198)	0.14
LGE, *n* (%)	48 (9.8)	6 (6.1)	42 (10.7)	2.367 (0.802–6.991)	0.20		
≥ 2 ectopic sites, *n* (%)	141 (28.7)	25 (25.5)	116 (29.5)	0.960 (0.524–1.756)	0.89		
Under AAD, *n* (%)	361 (73.5)	73 (74.5)	288 (73.3)	0.941 (0.539–1.642)	0.79		
LVEF, % (mean ± SD)	57 (50–60)	55 (45–60)	58 (50–60)	1.025 (1.001–1.050)	0.04	1.010 (0.978–1.043)	0.56
PVC number, *n* (median ± SD)	20 275 (12 761–27 910)	20 993 (10 454–25 346)	20 116 (13 110–28 805)	1.000 (1.000–1.000)	0.36		
Few PVC during procedure, *n* (%)	78 (15.9)	11 (11.2)	67 (17.0)	1.996 (0.890–4.473)	0.12		
Plus pace mapping, *n* (%)	296 (60.3)	64 (65.3)	232 (59.0)	0.762 (0.459–1.265)	0.26		
Left-sided PVC, *n* (%)	258 (52.5)	45 (45.9)	213 (54.1)	0.799 (0.485–1.316)	0.38		
Image integration, *n* (%)	92 (18.7)	25 (25.5)	67 (17.0)	0.632 (0.350–1.140)	0.13		
ICE, *n* (%)	36 (7.3)	7 (7.1)	29 (7.4)	0.861 (0.347–2.136)	0.87		
Catheter for unipolar signal, *n* (%)	242 (49.3)	29 (29.6)	211 (53.7)	2.732 (1.614–4.623)	<0.001	2.652 (1.275–5.518)	0.009
Power, W (mean ± SD)	37 ± 8	37 ± 8	37 ± 8	1.006 (0.972–1.042)	0.73		
Ablation time, s (mean ± SD)	639 ± 612	746 ± 467	638 ± 737	1.000 (0.999–1.000)	0.24		
Recurrence during the waiting time, *n* (%)	108 (22)	41 (41.8)	67 (17.0)	0.263 (0.152–0.456)	<0.001	0.330 (0.162–0.672)	0.002
Multipolar group, *n* (%)	204 (41.5)	33 (33.7)	171 (43.5)	1.517 (0.954–2.413)	0.08		
CARTO, *n* (%)	420 (85.5)	80 (81.6)	340 (86.5)	0.667 (0.341–1.306)	0.24		
Ablation with CF catheter, *n* (%)	458 (93.3)	89 (90.8)	369 (93.9)	1.406 (0.554–3.571)	0.49		
Manual verification of the points, *n* (%)	306 (62.3)	58 (59.2)	248 (63.1)	1.091 (0.659–1.806)	0.66		

AAD, antiarrhythmic drug; CF, contact force; ICE, intracardiac echocardiography; LGE, late gadolinium enhancement; LVEF, left ventricle ejection fraction; PVC, premature ventricular contraction

There was no significant difference in ventricular arrhythmia freedom at midterm follow-up between higher- and lower-volume centres (82.4% vs. 78.6%, *P* = 0.22) concerning ventricular arrhythmia freedom (Supplementary material online).

Among patients who had recurrence during follow-up, a redo procedure was performed in 54% (36 patients out of 67), with multielectrode mapping being used in the majority of the redo cases (75%).

### Sensitive analysis for left-sided PVC

Baseline and procedural characteristics of left-sided PVC ablation are detailed in Supplementary material online, Results and *Tables S5* and *S6*.

**Figure 4:**
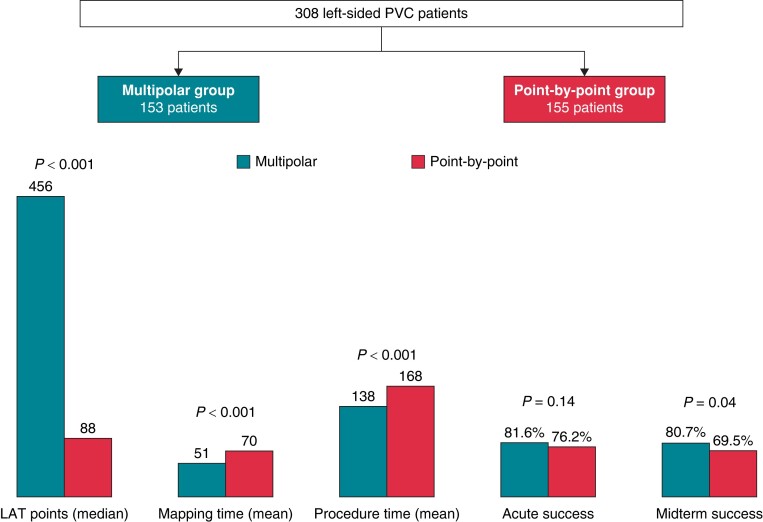
Left-sided PVC procedural data and acute and midterm outcomes.

The median number of points acquired in the Multipolar group was 456 (290–1032) during 51 ± 40 min of mapping compared with 88 (46–223) (*P* < 0.001) during 70 ± 58 min of mapping (*P* < 0.001) in the PbP group (*Table S6* and *Figure 4*). Conversely, in the Multipolar group, additional pace mapping (39.3% vs. 79.4%, *P* < 0.001) and manual verification of the annotated points (29.4% vs. 91.6%, *P* < 0.001) were less commonly performed. There were no differences regarding the utilization of a unipolar catheter (50.3% in the Multipolar group vs. 56.8% in the Control group, *P* = 0.23). Patients in the Multipolar group had shorter ablation time (629 ± 528 vs. 870 ± 930 s, *P* = 0.02) and shorter overall procedure time (138 ± 68 vs. 168 ± 67 min, *P* < 0.001) (Supplementary material online, *Table S6* and *Figure 4*).

Acute success was high and similar between groups (81.6% in Multipolar group vs. 76.2% in PbP group, *P* = 0.14), with a lower recurrence rate during the waiting period in patients receiving multipolar mapping (17% vs. 29.7%, *P* = 0.003) (*Figure 4*).

Of the 308 patients who had left-sided PVC ablation, 258 completed a minimum of 3 months of follow-up. Freedom from ventricular arrhythmia was higher in the Multipolar group (80.7% vs. 69.5%, *P* = 0.04) (*Figure 4*). After adjustment on all univariate predictors of the primary outcome, the use of a multielectrode mapping catheter was independently associated with freedom from recurrent PVC of left-sided origin [OR = 2.231 (95% CI 1.476–5.108), *P* = 0.02]. The use of a catheter for unipolar signal [OR = 2.848 (95% CI 1.611–6.697), *P* = 0.01] and ablation with a CF sensing catheter [OR = 4.623 (95% CI 1.776–8.245), *P* = 0.01] were also independent predictors of midterm efficacy (*Table 4*).

**Table 4:** Predictors of recurrence (>20% PVC burden at midterm follow-up) for left-sided PVC ablation

	Left PVC (*n* = 258)	PVC reduction <80% (*n* = 64)	PVC reduction ≥ 80% (*n* = 194)	Univariate model		Multivariate model	
				OR (95% CI)	P value	OR (95% CI)	*P* value
Male gender, *n* (%)	130 (50.4)	32 (50)	98 (50.5)	1.075 (0.926–1.097)	0.95		
Age, years [median (IQR)]	61 (50–70)	58 (51–68)	59 (49–69)	1.003 (0.980–1.026)	0.81		
Previous PVC ablation, *n* (%)	31 (12.0)	10 (15.6)	21 (10.8)	0.537 (0.211–1.362)	0.28		
Structural heart disease, *n* (%)	96 (37.2)	25 (39.1)	71 (36.6)	0.550 (0.259–1.170)	0.22		
LGE, *n* (%)	32 (12.4)	5 (7.8)	27 (13.9)	3.026 (0.668–13.707)	0.14		
≥ 2 ectopic sites, *n* (%)	82 (31.8)	24 (37.5)	58 (29.9)	1.584 (0.694–3.616)	0.09		
Under AAD, *n* (%)	180 (69.8)	44 (68.8)	136 (70.1)	0.661 (0.268–1.631)	0.67		
LVEF, % (mean ± SD)	51 ± 11	51 ± 10	52 ± 11	1.013 (0.981–1.047)	0.43		
PVC number, *n* (mean ± SD)	23 408 ± 15 933	19 609 ± 11 082	25 002 ± 18 105	1.000 (1.000–1.000)	0.07		
Few PVC during procedure, *n* (%)	36 (14.0)	10 (15.6)	26 (13.4)	0.755 (0.566–1.441)	0.35		
Plus pace mapping, *n* (%)	129 (50)	33 (51.7)	96 (49.5)	0.676 (0.330–1.386)	0.48		
Image integration, *n* (%)	41 (15.9)	13 (20.3)	28 (14.4)	0.583 (0.250–1.358)	0.28		
ICE, *n* (%)	28 (10.9)	6 (9.4)	22 (11.3)	1.003 (0.346–2.908)	0.89		
Catheter for unipolar signal, *n* (%)	131 (50.8)	22 (34.4)	109 (56.2)	2.448 (1.359–4.411)	0.01	2.848 (1.611–6.697)	0.01
Manual verification of the points	142 (55)	33 (51.6)	109 (56.2)	1.464 (0.614–1.800)	0.60		
Power, W (mean ± SD)	41 ± 9	40 ± 9	41 ± 8	1.010 (0.965–1.057)	0.67		
Ablation time, s median (IQR)	707 (540–990)	843 (622–1259)	680 (411–945)	1.000 (0.999–1.000)	0.41		
Recurrence during the waiting time, *n* (%)	54 (20.9)	19 (29.7)	35 (18)	0.521 (0.272–0.998)	0.05	0.411 (0.216–0.709)	0.04
Multipolar group, *n* (%)	130 (50.4)	25 (39.1)	105 (54.1)	1.840 (1.035–3.274)	0.04	2.231 (1.476–5.108)	0.02
CARTO system, *n* (%)	230 (89.1)	54 (84.4)	176 (90.7)	1.466 (1.170–2.275)	0.13		
Ablation with CF sensing catheter, *n* (%)	221 (85.7)	47 (73.4)	174 (89.7)	3.147 (1.528–6.481)	0.01	4.623 (1.776–8.245)	0.01

AAD, antiarrhythmic drug; CF, contact force; ICE, intracardiac echocardiography; LGE, late gadolinium enhancement; LVEF, left ventricle ejection fraction; PVC, premature ventricular contraction

## Discussion

This multicentre international study describes the outcome of PVC ablation guided by two different mapping strategies in a large real-world cohort: one guided by high-density mapping using a dedicated multipolar catheter vs. one employing traditional point-by-point mapping with the ablation catheter. The main findings are: (i) multipolar mapping was preferred in procedures of higher anticipated complexity, such as those from the coronary cusps, the LV summit, or the papillary muscles, and in more complex patients, such as those with more structural heart disease and late gadolinium enhancement on cardiac MRI; (ii) as expected, the use of a multipolar catheter allows a higher number of LAT points per map, faster mapping, and procedure times; however, acute success or freedom from recurrence during follow-up is in general high, regardless of the mapping strategy; (iii) notwithstanding, in the specific subgroup of patients having left-sided PVC ablation, the use of multipolar mapping may not only reduce procedure times, but also potentially reduce the risk of recurrence during follow-up; this hypothesis can only be properly tested in a randomized study.

The use of dedicated multipolar mapping catheters with small, tightly spaced electrodes facilitates high-resolution mapping due to their small field of view and shorter EGG duration recording translating into a more accurate local time annotation.^5,11,12^ In agreement with previous reports,^5,6^ our study highlights the benefits of multipolar catheters for simultaneous LAT point acquisition with a single PVC, for more rapid target definition, shorter contact mapping times, and thus reduced overall procedure times. This is particularly advantageous in patients who present with few intraprocedural PVCs and may help explain the lower requirement for additional pace mapping when using multipolar catheters, as shown is this study. One possible limitation associated with the use of multielectrode mapping is the induction of ectopic beats.^13^ However, in our large real-world cohort, only in 2 out of 592 patients a shifting was required from the multipolar to the ablation catheter.

This study was not designed to compare multipolar with PbP mapping. However, it provides a real-life assessment of outcomes of PVC ablation performed in multiple centres by a reasonable number of operators and using two different mapping strategies. Our results suggest that the choice of mapping strategy is not necessarily decisive for the outcome of the ablation, as overall acute and midterm success rates are high regardless of the mapping technique, with no significant differences between groups. The success rates described in our cohort are in line with those described in previous studies.^10,14–17^ However, a recent study from our research group^6^ had reported a 1-year efficacy of 89.7% with the multipolar catheter compared to just 70.6% when mapping was guided by the ablation catheter. Several reasons may help explain why the results of the present study differ: (i) this study did not require a standardized protocol for PVC ablation, contrary to the abovementioned one performed by Sousa *et al.*,^6^ where PVC ablation in the multipolar group followed a prespecified protocol where mapping was guided by the combined use of the PentaRay^TM^ catheter and the PMF software; (ii) compared to that study,^6^ where all procedures were carried out with a unipolar catheter, in this study, there was less use of a unipolar catheter in the Multipolar group, which may have had an impact on our results since the use of an unipolar catheter was indeed associated with improved outcome. The unipolar catheter has an important role since it reduces noise, thus facilitating the correct annotation of the clinical PVC and avoiding the incorrect inclusion of mechanical PVCs into the activation map; (iii) in the present study, there was less manual verification of LAT points in the Multipolar group compared to patients in the PbP group. Having more LAT points does not by itself assure improved success rates, since the quality of the electrograms and mapping are equally important. This was demonstrated in our previous study,^6^ where we systematically used a catheter for unipolar signal, which increased the accuracy of the LAT map, and, before ablation, bipolar and unipolar deflections of the earliest LAT points were always reviewed; iv) finally, in the present study, freedom from ventricular arrhythmia in the PbP group was higher than in the study of Sousa *et al.*,^6^ which can probably be explained by the lower number of left-sided PVC ablations in the former.

The marked differences between multipolar and PbP groups and the higher overall complexity of procedures in the former suggest that any comparison between these two strategies should be interpreted cautiously. In fact, multipolar mapping was more frequently used in complex procedures (such as left-sided ablations), and there was a trend towards more frequent structural heart disease and late gadolinium enhancement in these patients. As a result, the apparent lack of difference in outcomes between groups may actually suggest that a multipolar approach may be beneficial, as we would have expected worse outcomes in patients submitted to more complex procedures (i.e. those in the multipolar group). Notwithstanding, the use of a multipolar catheter was an independent predictor of success in left-sided PVC ablations. Since left-sided PVCs can originate from several close locations, it is plausible that the higher number of points collected in short time with the multielectrode catheter can more easily direct us to the earliest activation area, which may also explain the lower recurrence rate during the waiting period seen in patients of the Multipolar group. This is particularly important, given that recurrence during the waiting period was an independent predictor of midterm recurrence for the overall population cohort and also left-sided ablation specifically. Also, by acquiring more points, multielectrode mapping may partially mitigate the displacement that occurs when mapping PVCs to their location in sinus rhythm. Contrarily, the lack of accuracy when performing PbP mapping with the ablation catheter may result in RF ablation delivered at a slightly displaced location, which temporarily suppresses the arrhythmia, but does not eliminate the source of origin permanently.^18,19^ Conversely, PVCs originating from the right ventricular outflow tract may not require such detailed mapping, as this is a more well-defined area with fewer possible alternative locations.

Despite a non-negligible complication rate, severe complications occurred in a minority of patients (2.0%), in line with other large studies.^14,16^ Importantly, although left-sided and epicardial PVCs were performed more frequently in the multipolar group, no significant difference in complication rates was observed between groups.

The cost of adding a multipolar mapping catheter should be weighed against its benefits. The use of the multipolar catheter reduces procedure duration and therefore allows for higher patient turnover. In light of the results of this study, it may be argued that the additional cost may be justifiable in patients with left-sided PVC. However, a cost-effectiveness assessment was beyond the scope of this study.

In summary, this retrospective, multicentre study of PVC ablation provides a real-world assessment of two different mapping strategies: multipolar mapping with a multielectrode catheter and point-by-point mapping with the ablation catheter. In general, mapping with a multielectrode catheter does not necessarily improve the acute or midterm outcomes of PVC ablation, although a benefit in left-sided PVC ablation may be possible. Conversely, multielectrode mapping allows for shorter procedures without increased complication rates.

## Limitations

This study has some limitations. Firstly, this was a retrospective assessment, and, therefore, important baseline and procedural differences exist between groups. Patients in the multipolar group underwent more often left-sided procedures. For this reason, lower success rates could have been expected *a priori* in the multipolar group. Therefore, the trend towards higher success rate may in fact suggest a possible benefit of multipolar mapping, yet causality would require a prospective randomized study. We highlight, however, that this study was not designed to compare multipolar vs. PbP mapping. Secondly, the PentaRay^TM^ catheter was used for the majority of patients in the multipolar group. This precludes an assessment on whether a specific multielectrode catheter may be more advantageous than others. Nevertheless, this study did not aim to compare different mapping catheters or mapping systems. Thirdly, we also acknowledge that follow-up was heterogeneous in terms of duration and assessment of post-procedural PVC burden. We recognize that PVC burden can vary on a daily basis, and, therefore, more frequent 24 h Holters or even long-term monitors would better reflect the impact of the mapping strategy. However, the majority of PVC recurrences was evident already in the very early post-procedural phase or within the first 24 h. In general, later recurrences after 3 months following successful PVC ablation are uncommon. Also, there were no significant differences in the follow-up duration between both groups. Lastly, there is no standard accepted definition of successful ablation in terms of overall reduction in PVC burden; hence, our results can differ from other studies using other definitions.

## Conclusions

PVC ablation can be performed with high success rates, irrespective of whether a multipolar catheter is used. However, multipolar mapping allows for faster procedures and may be advantageous for reducing recurrences of left-sided PVC.

## Supplementary Material

euae148_Supplementary_Data

## Data Availability

The data underlying this article will be shared on reasonable request to the corresponding author.
