# Supplementary material for: Real-world assessment of multipolar and point-by-point mapping for premature ventricular contraction ablation
Source: Europace. 2024 May 31;26(6):euae148. doi: 10.1093/europace/euae148 (PMC11179105; doi:10.1093/europace/euae148)

**SUPPLEMENTAL METHODS**

**SUPPLEMENTAL RESULTS**

***Sensitive analysis for left-sided PVC***

The main baseline characteristics are detailed in **Table S-5**. Patients in the Multipolar group had lower median LVEF (50% vs. 55%, p<0.001) and more structural heart disease (45.8% vs 35.5%, p=0.03).

***Procedural characteristics***

The majority of the centers used the CARTO system, being PentaRay^TM^ the multielectrode catheter most frequently used (73.2%). (**Table S-6**) In the Multipolar group, ICE (16.3% vs. 1.3%, p<0.001) and cardiac image integration (34.6% vs. 5.2%, p<0.001) were significantly more frequently used than in the PbP group, whereas coronary angiography (13.1% vs. 36.8%, p<0.001) was less commonly performed.

***Sensitive analysis for high-volume PVC center***

Of the 10 centers included in this study, four performed more than 10 PVC ablations per year and were considered higher-volume PVC ablation centers. A total of 396 patients were treated in these four higher-volume centers, whereas 196 patients were treated in the remaining six, lower-volume centers.

There were no significant differences in the incidence of the primary outcome between higher-volume and lower-volume centers (82.4% vs. 78.6%, respectively, p=0.22). Multipolar mapping had similar outcome in higher (84.1%) and lower-volume centers (82.6%).

**SUPPLEMENTAL TABLE**

**Table S-1 –** Structural heart disease in the Multipolar and PbP groups.

|  | **All Patients**  **(n= 592)** | **Multipolar Group**  **(n=248)** | **Point-by-Point Group**  **(n=344)** |
| --- | --- | --- | --- |
| **Coronary artery disease, n (%)** | 64 (10.8) | 24 (9.7) | 40 (11.6) |
| **Hypertrophic cardiomyopathy, n (%)** | 1 (0.2) | 0 | 1 (0.3) |
| **Congenital heart disease, n (%)** | 6 (1.0) | 4 (1.6) | 2 (0.6) |
| **Dilated cardiomyopathy, n (%)** | 80 (13.5) | 45 (18.1) | 35 (10.2) |
| **Valvular heart disease, n (%)** | 9 (1.5) | 6 (2.4) | 3 (0.9) |
| **Arrhythmogenic right ventricular cardiomyopathy, n (%)** | 1 (0.2) | 0 | 1 (0.3) |
| **Myocarditis, n (%)** | 2 (0.4) | 2 (0.8) | 0 |

**Table S-2 –** PVC location in both groups.

|  | **All Patients**  **(n= 592)** | **Multipolar Group**  **(n=248)** | **Point-by-Point Group**  **(n=344)** | **P value** |
| --- | --- | --- | --- | --- |
| **Left-sided, n (%)** | 308 (52) | 153 (61.7) | 155 (45.3) | <0.001 |
| **Right-sided, n(%)** | 284 (48) | 95 (38.3) | 189 (54.9) | <0.001 |
| **RVOT, n (%)** | 282 (47.6) | 95 (38.3) | 187 (54.4) | <0.001 |
| **Other right ventricular origin, n (%)** | 2 (0.3) | 0 | 2 (0.6) | 0.30 |
| **LVOT, n (%)** | 81 (13.7) | 34 (13.7) | 47 (13.6) | 0.88 |
| **Coronary cusps, n (%)** | 61 (9.8) | 31 (12.5) | 30 (8.7) | 0.002 |
| **Aortic commissures, n (%)** | 22 (3.7) | 8 (3.2) | 14 (4.1) | 0.57 |
| **Papillary muscle, n (%)** | 23 (3.9) | 14 (5.6) | 9 (2.6) | 0.06 |
| **LV summit, n (%)** | 39 (6.6) | 24 (9.7) | 15 (4.4) | 0.004 |
| **Great cardiac vein, n (%)** | 3 (0.5) | 2 (0.8) | 1 (0.3) | 0.39 |
| **Aorto-mitral continuity, n (%)** | 26 (4.4) | 14 (5.7) | 12 (3.5) | 0.09 |
| **Mitral annulus, n (%)** | 19 (3.2) | 9 (3.6) | 10 (2.9) | 0.63 |
| **Scar-related, n (%)** | 15 (2.5) | 8 (3.2) | 7 (2.0) | 0.37 |
| **Epicardial site, n (%)** | 9 (1.6) | 5 (2.3) | 4 (1.2) | 0.29 |
| **Other site, n (%)** | 10 (1.7) | 4 (1.6) | 6 (1.7) | 0.85 |

Abbreviations:

LV – left ventricular, LVOT – left ventricular outflow tract, RVOT – right ventricular outflow tract;

**Table S-3 –** Predictors of acute recurrence during PVC ablation.

|  | | | | *Univariate model* | | *Multivariate model* | |
| --- | --- | --- | --- | --- | --- | --- | --- |
|  | All Patients  (n=592) | No acute success  (92) | Acute success  (n=500) | *OR, (95% CI)* | *P* value | *OR, (95% CI)* | *P* value |
| Male gender, n (%) | 273 (46) | 39 (42) | 234 (47) | 1.011 (0.945 - 1.080) | 0.76 |  |  |
| Age, years (median [IQR]) | 54 (41-65) | 57 (42-68) | 53 (40-64) | 0.987 (0.973 - 1.002) | 0.91 |  |  |
| Previous PVC ablation, n (%) | 67 (11.3) | 7 (7.6) | 60 (12) | 1.546 (.682 - 3.505) | 0.29 |  |  |
| Structural heart disease, n (%) | 173 (29.2) | 30 (32.6) | 143 (28.6) | 0.729 (0.446 - 1.191) | 0.21 |  |  |
| LGE, n (%) | 51 (8.6) | 15 (16.3) | 36 (7.2) | 0.320 (0.162 - 0.629) | <0.001 | 0.414 (0.130 – 1.312) | 0.13 |
| ≥2 ectopic sites, n (%) | 129 (21.8) | 14 (15.2) | 115 (23) | 1.369 (0.711 - 2.635) | 0.35 |  |  |
| Under AAD, n (%) | 419 (70.8) | 63 (68.5) | 356 (71.2) | 0.845 (0.497 - 1.439) | 0.54 |  |  |
| LVEF, % (median [IQR]) | 55 (50-60) | 56 (46-62) | 55 (50-60) | 1.003 (0.979 - 1.026) | 0.83 |  |  |
| PVC number, n median (IQR) | 20197 (12147-29275) | 21000 (12720-30000) | 20000 (12055-28793) | 1.000 (1.000 - 1.000) | 0.41 |  |  |
| Few PVC during procedure, n (%) | 88 (17.4) | 14 (15.2) | 74 (14.8) | 0.921 (0.483 - 1.756) | 0.80 |  |  |
| Left-sided PVC, n (%) | 308 (52) | 54 (58.7) | 254 (50.8) | 1.450 (1.003 - 2.836) | 0.16 |  |  |
| Plus pace-mapping, n (%) | 398 (67.2) | 54 (58.7) | 344 (68.8) | 1.588 (0.994 - 2.536) | 0.05 | 1.090 (0.380 – 3.126) | 0.87 |
| Image integration, n (%) | 94 (15.9) | 14 (15.2) | 80 (16) | 0.980 (0.527 - 1.822) | 0.95 |  |  |
| ICE, n (%) | 35 (5.9) | 8 (8.7) | 27 (5.4) | 0.521 (0.228 - 1.193) | 0.12 |  |  |
| Catheter for unipolar signal, n (%) | 295 (49.8) | 34 (37) | 261 (52.2) | 1.361 (0.828 - 2.238) | 0.13 |  |  |
| Power, Watts (mean±SD) | 37 ±8 | 37 ± 10 | 37 ± 8 | 1.002 (0.970 - 1.034) | 0.92 |  |  |
| Ablation time, sec (mean±SD) | 639 ± 612 | 804 ± 640 | 613 ± 605 | 1.000 (.999 - 1.000) | 0.027 | 1.000 (0.999-1.000) | 0.12 |
| Recurrence during the waiting time, n (%) | 126 (22.5) | 57 (62) | 69 (13.8) | 0.043 (0.023 - 0.081) | <0.001 | 0.023 (0.008 - 0.067) | <0.001 |
| Manual verification of the points, n (%) | 386 (67) | 44 (47.8) | 342 (68.4) | 1.705 (1.049 - 2.772) | 0.03 | 1.752 (0.456-6.737) | 0.41 |
| Multipolar group, n (%) | 248 (41.9) | 28 (30.4) | 220 (44) | 1.846 (1.040 - 2.606) | 0.032 | 2.411 (0.869 - 6.691) | 0.09 |
| CARTO system, n (%) | 476 (82.6%) | 60 (71.4%) | 416 (84.6%) | 0.457 (0.268 - .778) | 0.003 | 1.929 (0.574 – 6.484) | 0.29 |

Abbreviations:

AAD – antiarrhythmic drug, ICE – intracardiac echocardiography, LGE – late gadolinium enhancement, LVEF – left ventricle ejection fraction, PVC – Premature Ventricular Contraction;

**Table S-4 –** Complications occurred in both groups, according to their severity.

| **Complications** | **Multipolar group** | **Point-by-Point group** |
| --- | --- | --- |
| **Major complications** | | |
| **Death, n** | - | - |
| **Stroke, n** | 1 | 1 |
| **Other systemic emboli, n** |  | 1 |
| **Cardiac tamponade, n** | 2 | 1 |
| **Vascular injury, n** | 2 | 3 |
| **Damage to valves or coronary arteries, n** | 1 |  |
|  | | |
| **Minor complications** | | |
| **Vascular complications not requiring intervention/surgery, n** | 2 | 3 |
| **Pericardial effusion/pericarditis, n** | 6 | 3 |
| **AV block II or III, n** | 1 |  |

**Table S-5 –** Baseline characteristics for left-sided PVC.

|  | **Left PVC**  **(n=308)** | **Multipolar Group**  **(n=153)** | **Point-by-Point Group**  **(n=155)** | **P value** |
| --- | --- | --- | --- | --- |
| **Age, years (median [IQR])** | 61 (50 - 70) | 62 (51 - 70) | 60 (49 - 69) | 0.59 |
| **Male gender, n (%)** | 161 (52.3) | 82 (53.6) | 79 (51) | 0.58 |
| **Previous PVC ablation, n (%)** | 42 (13.6) | 20 (13.1) | 22 (14.1) | 0.71 |
| **Structural Heart Disease, n (%)** | 125 (40.6) | 70 (45.8) | 55 (35.5) | 0.03 |
| **CMR, n (%)** | 121 (39.3) | 57 (37.3) | 64 (41.3) | 0.19 |
| **LGE, n (%)** | 33 (10.7) | 18 (11.8) | 15 (9.7) | 0.37 |
| **≥2 PVC ectopies, n (%)** | 83 (27) | 40 (26.1) | 43 (27.7) | 0.89 |
| **Under AAD, n (%)** | 215 (69.8) | 105 (68.6) | 110 (71) | 0.49 |
| **LVEF, % (median [IQR])** | 55 (43 - 60) | 50 (40 - 59) | 55 (50 - 60) | <0.001 |
| **PVC number, median (IQR)** | 21000 (13606 - 29875) | 20560 (12580 - 30235) | 21505 (15084 - 28763) | 0.57 |
| **% PVC, (mean±SD)** | 23 ± 11 | 23 ± 11 | 22 ± 10 | 0.83 |

Abbreviations:

AAD – antiarrhythmic drug, CMR – cardiac magnetic resonance, LGE – late gadolinium enhancement, LVEF – left ventricle ejection fraction, PVC – Premature Ventricular Contraction;

**Table S-6 –** Left-sided PVC procedural parameters.

|  | **Left PVC**  **(n=308)** | **Multipolar Group**  **(n=153)** | **Point-by-Point Group**  **(n=155)** | **P value** |
| --- | --- | --- | --- | --- |
| **Mapping system, n (%)** |  |  |  | 0.29 |
| CARTO, n (%) | 258 (83.8) | 124 (81) | 134 (86.5) |  |
| NavX/Ensite, n (%) | 50 (16.2) | 26 (17) | 17 (10.9) |  |
| **Catheter for unipolar signal, n (%)** | 165 (53.4) | 77 (50.3) | 88 (56.8) | 0.23 |
| **Image integration, n (%)** | 61 (19.8) | 53 (34.6) | 8 (5.2) | <0.001 |
| **ICE, n (%)** | 27 (8.8) | 25 (16.3) | 2 (1.3) | <0.001 |
| **Retrograde access, n (%)** | 222 (72.1) | 108 (70.6) | 114 (73.5) | 0.50 |
| **Coronary angiography, n (%)** | 77 (25) | 20 (13.1) | 57 (36.8) | <0.001 |
| **Use of ablation tools (AI, LSI)** | 163 (52.9) | 132 (86.3) | 31 (20) | <0.001 |
| **Patients with few PVC during the procedure, n (%)** | 43 (14) | 20 (13.1) | 23 (14.8) | 0.71 |
| **Pace mapping, n (%)** | 183 (26.9) | 60 (39.2) | 123 (79.4) | <0.001 |
| **Points acquired, n (median [IQR])** | 231 (76 - 549) | 456 (290 - 1032) | 88 (46 - 223) | <0.001 |
| **Manual verification of the points, n (%)** | 187 (60.7) | 45 (29.4) | 142 (91.6) | <0.001 |
| **Power, Watts (mean±SD)** | 41 ± 9 | 43 ± 10 | 39 ± 8 | 0.001 |
| **Mapping time, min (mean±SD)** | 60 ± 51 | 51 ± 40 | 70 ± 58 | <0.001 |
| **CF sensing catheter, n (%)** | 258 (83.8) | 131 (85.6) | 127 (81.9) | 0.32 |
| **Ablation time, sec (mean±SD)** | 740 ± 749 | 629 ± 528 | 870 ± 930 | 0.02 |
| **Procedure time, min (mean±SD)** | 153 ± 67 | 138 ± 68 | 168 ± 67 | <0.001 |

Abbreviations:

AI – Ablation Index, CF – contact force, ICE – intracardiac echocardiography, PVC – Premature Ventricular Contraction, LSI – Lesion Size Index;

**SUPPLEMENTAL FIGURE**

**Figure S-1-** Acute recurrences observed in the Multipolar group.


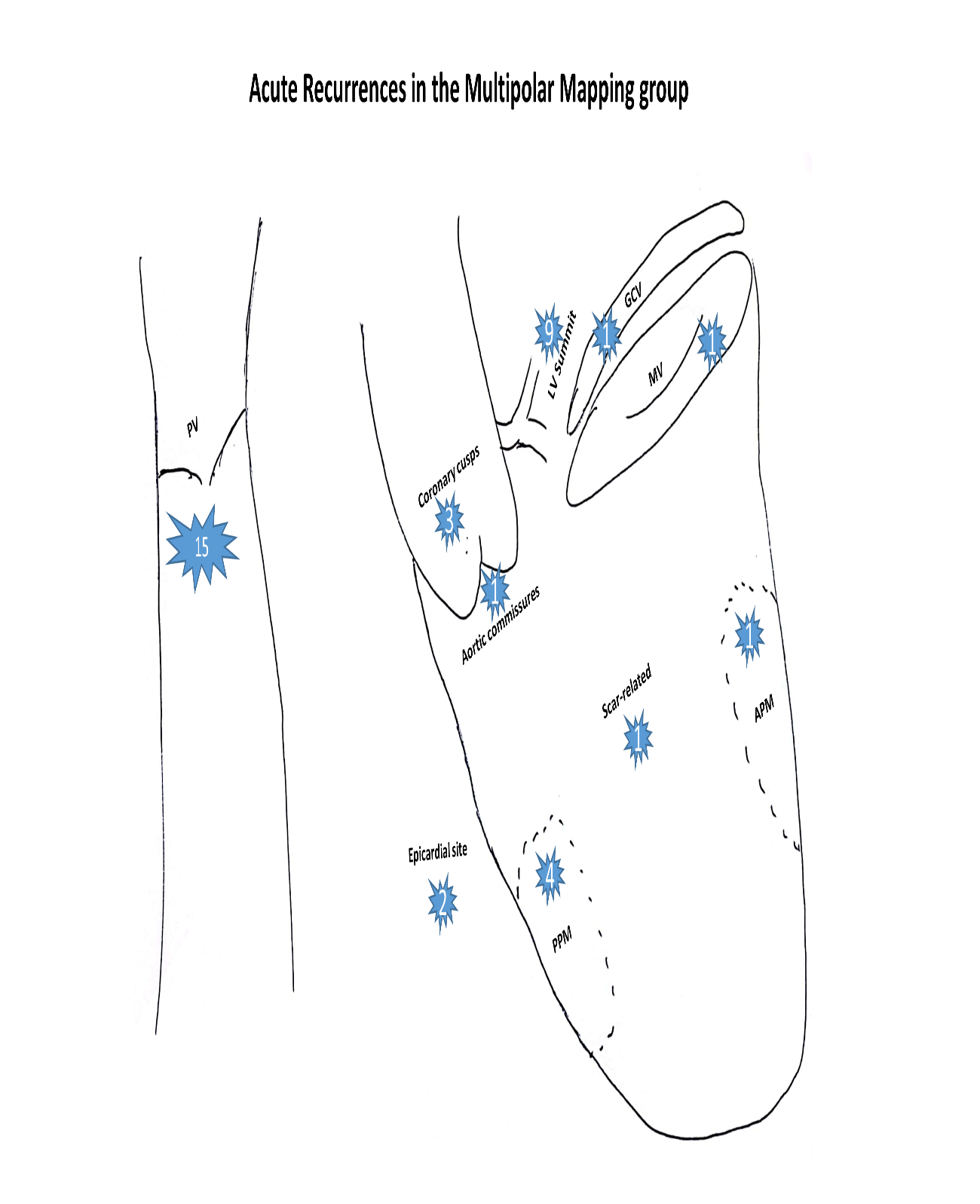

Supplement: euae148_Supplementary_Data [file euae148_supplementary_data.docx]
